# The effect of emotion intensity on time perception: a study with transcranial random noise stimulation

**DOI:** 10.1007/s00221-023-06668-9

**Published:** 2023-07-21

**Authors:** Antonino Visalli, Chiara Begliomini, Giovanna Mioni

**Affiliations:** 1 IRCCS San Camillo Hospital, Venice, Italy; 2 Department of General Psychology, University of Padova, 35131 Padua, Italy; 3 Padova Neuroscience Center, Padua, Italy

**Keywords:** Time perception, Emotion, Random noise stimulation, tRNS, Orbitofrontal cortex

## Abstract

Emotional facial expressions provide cues for social interactions and emotional events can distort our sense of time. The present study investigates the effect of facial emotional stimuli of anger and sadness on time perception. Moreover, to investigate the causal role of the orbitofrontal cortex (OFC) in emotional recognition, we employed transcranial random noise stimulation (tRNS) over OFC and tested the effect on participants’ emotional recognition as well as on time processing. Participants performed a timing task in which they were asked to categorize as “short” or “long” temporal intervals marked by images of people expressing anger, sad or neutral emotional facial expressions. In addition, they were asked to judge if the image presented was of a person expressing anger or sadness. The visual stimuli were facial emotional stimuli indicating anger or sadness with different degrees of intensity at high (80%), medium (60%) and low (40%) intensity, along with neutral emotional face stimuli. In the emotional recognition task, results showed that participants were faster and more accurate when emotional intensity was higher. Moreover, tRNS over OFC interfered with emotion recognition, which is in line with its proposed role in emotion recognition. In the timing task, participants overestimated the duration of angry facial expressions, although neither emotional intensity not OFC stimulation significantly modulated this effect. Conversely, as the emotional intensity increased, participants exhibited a greater tendency to overestimate the duration of sad faces in the sham condition. However, this tendency disappeared with tRNS. Taken together, our results are partially consistent with previous findings showing an overestimation effect of emotionally arousing stimuli, revealing the involvement of OFC in emotional distortions of time, which needs further investigation.

## Introduction

Emotional facial expressions are fundamental for social interactions (Frith 2009). It is also recognized that emotional stimuli can distort our sense of time (Droit-Volet and Meck 2007; LaBar and Meck 2016): for example, when we are sad and or happy, we have the feeling that the flow of time slows down or speeds up. The present study investigates the effect of facial emotional stimuli of anger and sadness on time perception and the possibility that the orbitofrontal cortex (OFC) could play a role in emotional recognition and emotional distortions of time perception by applying transcranial electric stimulation over OFC.

The effect of emotional stimuli (i.e., facial expression of emotions; Droit-Volet and Gil 2009) on time perception is most often explained based on an internal-clock model (Church and Meck 1984; Treisman 1963; Zakay and Block 1995). The model predicts that the subjective time comes from the number of pulses produced by a pacemaker and stored in an accumulator during the event to be timed. The more pulses accumulated, the longer the duration is estimated (Gibbon et al. 1984). Empirical findings have shown that an increase in the level of arousal increases the speed of the pacemaker (Droit-Volet and Meck 2007; Lake et al. 2016); previous studies showed that emotional pictures generating high arousal led to greater overestimation of time, compared to emotional stimuli generating less arousal (Brunot and Niedenthal 2004; Droit-Volet and Gil 2009; Droit-Volet et al. 2011; Seelam and O’Brien, 2011; Noulhiane et al. 2007; Tipples 2008).

Emotional facial expressions are widely regarded as the most powerful means for humans to communicate their emotions to others (Ekman 1982; 1984). Previous research has revealed that facial expressions depicting anger, fear, sadness, and happiness lead to an overestimation of perceived duration compared to neutral stimuli (Droit-Volet and Meck 2007; Gil and Droit-Volet 2011). This overestimation has been linked to the readiness to establish social connections with others (Fayolle and Droit-Volet 2014; Lake et al. 2016; Schirmer 2011). Action readiness refers to individuals' predisposition to engage in social relationships by either approaching or avoiding others. For example, when interacting with someone who displays anger, signalling a potential for aggression, arousal is automatically triggered. This heightened state of arousal serves to prepare the body for a rapid response to a potentially threatening event. Therefore, in the presence of an angry face announcing the possibility of a threat, the clock rates would thus increase; the more rapidly time elapses the more quickly the organism is ready to react, flee or attack (Watts and Sharrock 1984; Bar-Haim et al. 2010; Buetti and Lleras 2012). On the other hand, sadness is generally recognized as being less arousing than anger, even if inducing a general activation of the individual (Droit-Volet et al. 2004; Fayolle and Droit-Volet 2014; Gil and Droit-Volet 2011). In this vein, we can hypothesize that the perception of a sad face increases the level of arousal of the perceiver in order to aid. Several studies have investigated and compared the effects of angry and sad emotional expressions on time perception (Droit-Volet et al. 2004; Fayolle and Droit-Volet 2014; Gil and Droit-Volet 2011), reporting a tendency to overestimate the duration of angry/sad faces in comparison to neutral expressions. However, the tendency of overestimation seems to be greater for angry rather than sad faces (Droit-Volet et al. 2004; Gil and Droit-Volet 2011), suggesting how the impact of emotional expressions on time perception can vary according to the specific emotional content and its corresponding arousal level.

It should be acknowledged that, apart from the arousal-based model of emotional distortion of time perception, other emotional aspects have been identified to influence time perception. For instance, research has demonstrated that emotional valence can interact with arousal to affect time perception. In a pioneering study by Angrilli et al. (1997), both arousal (high vs low intensity) and valence (positive vs negative) were controlled, revealing that high arousal images led to an overestimation of durations for negative stimuli compared to positive stimuli. Conversely, low arousal images resulted in a greater underestimation of durations for negative stimuli compared to positive stimuli. The authors concluded that attentional factors impact temporal judgments when low-arousing stimuli are presented, while emotion-driven mechanisms operating at the level of the internal clock are involved when high-arousing stimuli are presented. Some studies have explored also the role of motivation in time perception, providing insights into how different motivational states can influence our perception of time. These studies consistently indicate that approach-motivated affects tend to speed up the perception of time, whereas withdrawal-motivated affects tend to slow it down (Gable et al. 2022).

How our brain connects the perceptual representation of a facial expression to one’s conceptual knowledge of the emotion signalled by the facial expression has been investigated by Adolphs (2002), suggesting a key role for the OFC. Indeed, the OFC appears to be critical for recognising emotions through multiple sensory modalities (Kringelbach and Rolls 2004; Heberlein et al. 2008). Goodkind and colleagues (2012) in a functional Magnetic Resonance Imaging (fMRI) study explored the contribution of OFC in a dynamic emotion tracking task, involving both patients with various neurodegenerative diseases and healthy participants. Results showed that low tracking accuracy was primarily associated with grey matter loss in the right lateral OFC, confirming the critical role of this region in emotion recognition across time. Other patients’ studies have also shown that OFC damage is associated with an impaired ability to categorize or rate the intensity of facial expressions (Blair et al. 2000; Monte et al. 2013; Heberlein et al. 2008; Hornak et al. 1996; Marinkovic et al. 2000). Consistently, the typical deficit is recognizing negative emotions (e.g., sadness, anger) as opposed to positive (e.g., happy) ones (Willis et al. 2014; see also Heberlein et al. 2008; Zald and Andreotti 2010, for reviews).

Transcranial Electric Stimulation (tES) techniques are widely used in neuroscience research to explore the causal role of the cortical area in cognitive functions (Jahanshahi and Rothwell 2000; Walsh 2000; Paulus 2011). These techniques deliver a low-intensity current through two scalp electrodes produced by a portable battery-powered stimulator, and induce a temporary modulation of cortical excitability. The low-intensity electrical field generated by electrical stimulation is subthreshold and does not induce action potentials in resting neurons but induces neuromodulation that produces immediate and lasting changes in brain function (Jahanshahi and Rothwell 2000; Walsh 2000; Paulus 2011). Willis et al. (2015) used anodal transcranial Direct Current Stimulation (tDCS), a type of tES, to enhance facial expression recognition of angry, disgusted, fearful, happy and sad in healthy adults with a forced-choice labelling task. The authors targeted the right OFC by applying anodal stimulation at electrode location FP2 according to the 10/20 system with the cathodal electrode positioned over the left parietal cortex (equivalent to location P3). Results showed that anodal tDCS boosts the recognition of emotional facial expression compared to neutral ones.

Here, we tested for the effects of facial emotions on perceived duration of presented stimuli using the temporal bisection procedure in which participants are asked to judge temporal intervals in comparison to two standard intervals previously memorised (Kopec and Brody 2010). Furthermore, we tested for the modulatory effect of tES on emotion recognition and on temporal distortions induced by emotional expressions. Following Willis and colleagues work (2015) we targeted the right OFC, but we opted for an alternative technique named transcranial Random Noise Stimulation (tRNS), which delivers current at random frequencies. In contrast to tDCS adopted by Willis and colleagues (2015), tRNS has no constraint of current flow direction as both current intensity and frequency vary randomly (ranging from 0.1 to 640 Hz). Interestingly, weaker sensory sensations are usually reported with tRNS, compared to tDCS (Ambrus et al. 2010). Therefore, the application of tRNS might better suit for placebo-controlled studies (Antal and Herman 2016; Fertonani et al. 2015). tRNS after-effects are intensity-dependent, and stimulation at 1.5 mA leading to excitability after-effect is comparable to that observed with anodal tDCS (Inukai et al. 2016; Moliadze et al. 2014); ; ;. It was suggested that tRNS might increase synchronisation of neural firing through amplification of sub-threshold oscillatory activity, which in turn reduces the amount of endogenous noise. Therefore, the effects of tRNS might be based on mechanisms such as stochastic resonance (Schwarzkopf et al. 2011; Stacey and Durand 2000; Inukai et al. 2016).

In the present work, two negative emotions, i.e., anger and sadness, have been tested. Each of these emotional expressions have the same affective valence (negative), but different levels of arousal (anger is high-arousing; sadness is low-arousing). We hypothesize negative emotions inducing temporal overestimation (Gil and Droit-Volet 2011), with greater overestimation when a high arousing stimulus is presented (i.e., anger). We also manipulated the degree of emotional intensity of both anger and sad faces to investigate the effect of arousal on temporal overestimation within each emotion. Considering the tRNS has been shown to enhance visual (Romanska et al. 2015) and emotional perception (Penton et al. 2017; Yang and Banissy 2017) we predict temporal overestimation when facial emotional stimuli of anger are presented being greater under active stimulation (random condition).

## Methods

### Participants

Twenty university students participated in the study (mean age = 23.95; SD = 1.70; range 21–29 years old; mean education = 17.75 years; SD = 1.59). They were all recruited and tested at the Department of General Psychology, University of Padova. All participants were right-handed, as defined by the Edinburgh Handedness Inventory (Oldfield 1971). Exclusion criteria included a history of neurological or psychiatric illness, pregnancy, and use of drugs or alcohol 24 h before the experimental session.

### Procedure

Participants were tested individually during two experimental sessions one day apart in which they performed the experimental tasks under tRNS or sham conditions. In each session, participants performed both temporal and emotional recognition tasks. The stimulation protocol across sections (tRNS or sham) and the order of tasks within sessions (temporal task or emotional recognition task) were counterbalanced between participants. At the end of each experimental session, participants performed the Sensation questionnaire to control for sensations experienced during stimulation (Fertonani et al. 2015). The active stimulation lasted 20 min, and was performed during the tasks’ execution. Each session (tRNS and sham) lasted approximately 1 h. The study was approved by the ethics committee of Area 17 approved protocol reference number: 3110) Department of General of Psychology, University of Padova (Italy) and conducted according to the Declaration of Helsinki (59th WMA General Assembly, Seoul, 2008).

### Experimental tasks

#### Emotional recognition task

**Fig. 1:**
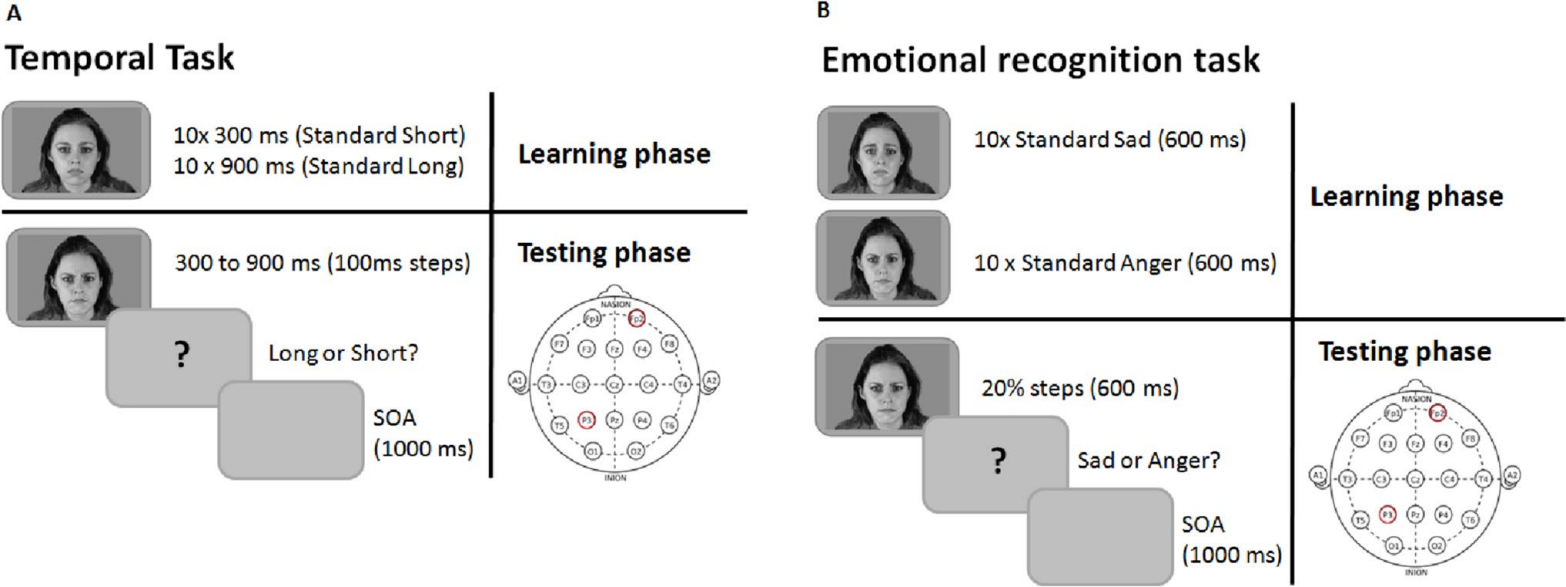
Graphical representation of temporal task (**A**) and emotional recognition task (**B**)

The session started with the learning phase in which participants were required to memorize the two standard emotional stimuli: standard sad and standard anger, presented 10 times for 600 ms centrally on the computer screen (Fig. 1B). After the learning phase, participants were required to judge different emotional stimuli and to decide if the comparison stimulus was more similar to the standard sad or the standard anger. The visual stimuli were facial emotional stimuli indicating anger or sadness but with different degrees of intensity from high (80%), medium (60%) and low (40%) intensity, along with neutral faces. Stimuli were selected from the Montreal Set of Facial Displays of Emotion (MSFDE; Beaupré and Hess 2005; Beaupré et al. 2000) morphing dataset. Facial emotional stimuli of two female and two male characters were selected (female 1 anger = 2 51, sad = 253; female 2 anger = 271, sad = 273; male 1 anger = 221, sad = 223 and male 2 anger = 231, sad = 233). The task consisted of 84 trials divided in three blocks. Within a block, each emotional stimulus was presented 4 times. After the response, a 1000 ms inter-trial interval was presented. No feedback was provided.

#### Temporal task

We used a time bisection task to tap time perception in our participants. The experimental session started with the learning phase in which participants were required to memorize the two standard durations: 300 ms (short standard) and 900 ms (long standard). Both standard durations were marked by neutral facial emotional stimuli presented 10 times (Fig. 1A). After the learning phase, participants were required to judge different temporal intervals (300, 400, 500, 600, 700, 800, and 900 ms) and to decide if the comparison interval was more similar to the short standard or the long standard. The visual stimuli were the same as those used for the emotional recognition task selected from MSFDE (Beaupré et al. 2000) morphing dataset. Each emotion (anger, sadness and neutral) and each intensity (high, medium, low and neutral) was presented for each temporal interval 4 times for a total of 196 stimuli in each block; participants performed three blocks for a total of 588 stimuli. After the response, there was a 1000-ms inter-trial interval. No feedback was provided.

### tRNS setup

Online high-frequency tRNS (tRNS, frequencies ranged from 100 to 640 Hz) was delivered using a battery-driven stimulator (BrainSTIM, EMS) through a pair of saline-soaked sponge electrodes and the electrodes were kept in place with plastic bandages. The tRNS consisted of a random current of 1.5 mA intensity with a 0 mA offset applied at random frequencies.

One electrode was placed over the right OFC (equivalent to location FP2, according to the international 10–20 EEG system, Oostenveld et al. 2001), with the reference electrode positioned over the left parietal cortex equivalent to location P3. After the electrodes montage, participants performed the training phase (see “time bisection task” and “Emotional recognition task” paragraphs) without active stimulation followed by the experimental phase (tRNS or sham condition). The total duration of stimulation was 20 min, and the entire session lasted approximately 1 h. The sham condition consisted of a 20 s ramp-up and 20-s ramp-down and 20 s of stimulation at the beginning and the end of the tasks. None of the participants reported pain experience following stimulation, and all participants included in the study completed all experimental sessions.

### Sensation experienced questionnaire

A questionnaire about the sensations experienced by participants during the two types of stimulations (tRNS, sham) was included (Fertonani et al. 2015). The questionnaire is composed of 7 questions concerning possible sensations experienced during stimulation; participants had to report how much they experienced that sensation on a scale ranging from not experienced = 0 to very much = 4. A total score was calculated by summing up the scores from all the questions included. Data are analysed using the Wilcoxon Matched-Pair Signed Ranks Test for Stimulation (tRNS vs sham).

### Statistical analyses

#### Emotional task

To assess how tRNS affects accuracy in facial emotion recognition, response accuracy was analyzed by conducting a Generalized Linear Mixed Model (GLMM) with logit link function (i.e., logistic regression) using the *glmer* function from the lme4 library (Bates et al. 2015). Trials with response times (RT) lower than 150 ms were considered anticipations and excluded. For this analysis, trials with Neutral faces were also excluded. The GLMM included emotion (two-level factor: sadness = -1, anger = 1), emotion intensity (continuous variable: low intensity = 0, medium intensity = 1, high intensity = 2), and stimulation (two-level factor: Sham = 0, tRNS = 1) and all their interaction terms as fixed effects. The random part included random intercepts for participants and facial emotional images. The random part did not include random slopes for the fixed effects, since their inclusion led to singularity.

In addition to the accuracy analysis, we also analysed RT from accurate trials using a linear mixed-effects model (LMM). The specification of the model was the same of the GLMM. To control for the impact of positive skewness in the distribution of RTs (in ms), all the analyses were performed on the inverse-transformed RTs (iRT), computed as -1000/RT (Brysbaert and Stevens 2018).

Finally, to verify whether there was a tendency in categorizing neutral faces as angry or sad and if this tendency was modulated by tRNS, the probability of “angry” responses to neutral faces was modelled using a GLMM (logit link function) including Stimulation (two-level factor: Sham = 0, tRNS = 1) as fixed effect and participants and facial emotional images as random intercepts. Trials with anticipations were excluded.

#### Temporal task

For each participant, the probability of “long” responses was modelled by means of a GLMM including interval duration (centred and scaled to facilitate model convergence), emotion (three-level factor), emotion intensity (continuous predictor), and stimulation (two-level factor) and their interactions as fixed effects. The random part included emotional images as random intercepts. The model was recursively fitted to change the reference levels of the factor variables and the coding of emotional intensity in order to extract the Bisection Point (BP) for each combination of the experimental variable (the BP can be described as the interval duration value corresponding to the 0.50 probability of “longer” responses on the *y*-axis; Grondin, 2008). For instance, to obtain the BP for angry faces at medium intensity and with tRNS, the variable Stimulation was coded as tRNS = 0 and Sham = 1, emotion was coded as anger = 0 and sadness = 1, intensity was coded as low = -1, medium = 0, high = 1. Since the model intercept corresponds to the probability of “long” response when all the predictors are at 0, the BP can be easily computed as—β_0_/β_1_, where β_0_ corresponds to the intercept and β_1_ is the slope of interval duration. This approach allowed us to consider all the variables together in the computation of BP and at the same to control for the random effects of the selected items (facial images). Trials with anticipations were excluded.

For the group-level analysis, we used the Constant Error (CE) as the dependent variable, which is defined as CE = neutral BP – emotional BP. Specifically, within each stimulation condition, each of the six emotional BP (two emotions by three intensity levels) was subtracted from the neutral BP. Using the constant error rather than the BP as the dependent variable allows the comparison of the perceived duration of the standard conditions (Neutral stimulus) compared to the two emotional stimuli (Azari et al. 2019). Defined this way (CE sadness = BP Neutral emotion–BP sadness; CE anger = BP Neutral emotion – BP anger) it facilitates the interpretation of the data as a higher CE value means more long responses (duration perceived as longer). The CE was analyzed by means of a Linear Mixed Model (LMM) which included emotion (two-level factor: sadness = -1, anger = 1), emotion intensity (continuous variable: low intensity = 0, medium intensity = 1, high intensity = 2), and stimulation (two-level factor: Sham = 0, tRNS = 1) and all their interaction terms as fixed effects. The random part included by-participant correlated random intercepts and slopes for the fixed effects.

## Results

### Emotional task

**Fig. 2:**
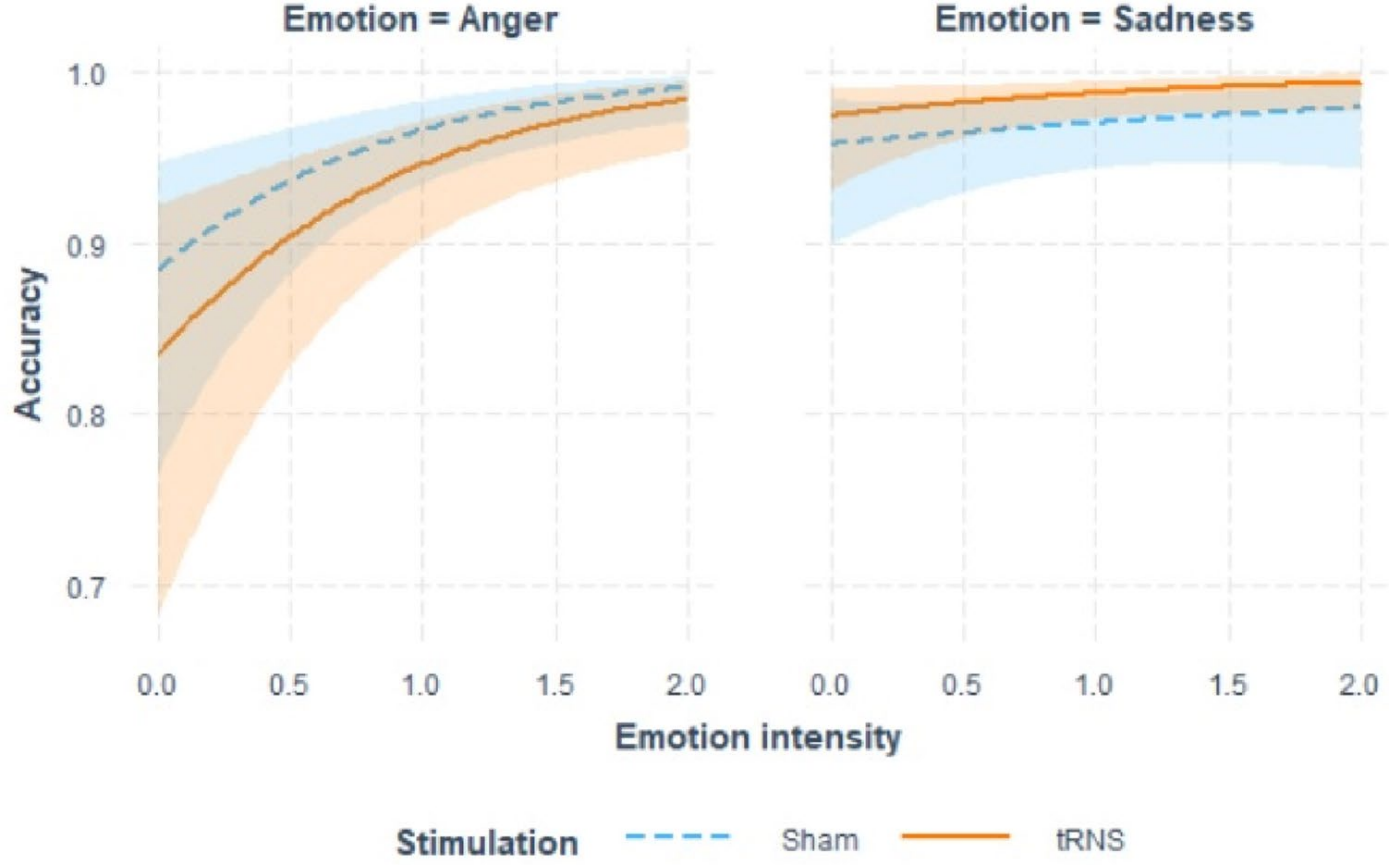
Interaction plot of the effects of emotion, emotion intensity and stimulation on recognition accuracy. The figure shows the conditional effect of emotion intensity on accuracy for sham (blue dashed lines) and tRNS (orange continuous line) stimulations separately for angry (left panel) and sad (right panel) emotional facial expressions. Shaded error bars indicate confidence intervals

Descriptive statistics for the emotional task are presented in Table 1. The results of the GLMM on recognition accuracy are presented in Table 2. Significant effects were found only for emotional intensity, with accuracy increasing as a function of emotional intensity. Although Fig. 2 seems to show a more pronounced emotional intensity effect for Angry expressions the interaction was not significant.

**Table 1:** Descriptive statistics of accuracy in the emotion recognition task. Low, medium and High indicate emotion intensity

Stimulation	Anger			Sadness		
	Low	Medium	High	Low	Medium	High
Sham						
Mean	0.80	0.94	0.99	0.94	0.96	0.97
(SD)	(0.12)	(0.09)	(0.03)	(0.12)	(0.08)	(0.07)
tRNS						
Mean	0.73	0.95	0.97	0.97	0.97	1.00
(SD)	(0.16)	(0.09)	(0.07)	(0.05)	(0.06)	(0.02)

**Table 2:** Summary output of the GLMM on emotion recognition accuracy

Predictors	Odds ratios	CI	*p*
(Intercept)	13.21	6.76–25.82	< 0.001
Emotion	0.58	0.32–1.05	0.071
Emotion intensity	2.33	1.38–3.92	**0.002**
Stimulation	1.05	0.64–1.73	0.836
Emotion × intensity	1.62	0.96–2.73	0.071
Emotion × stimulation	0.63	0.38–1.03	0.067
Intensity × stimulation	1.16	0.68–1.97	0.582
Emotion × intensity × stimulation	0.80	0.47–1.36	0.409

Bold *p*-values indicate statistical significance at α = 0.05

**Fig. 3:**
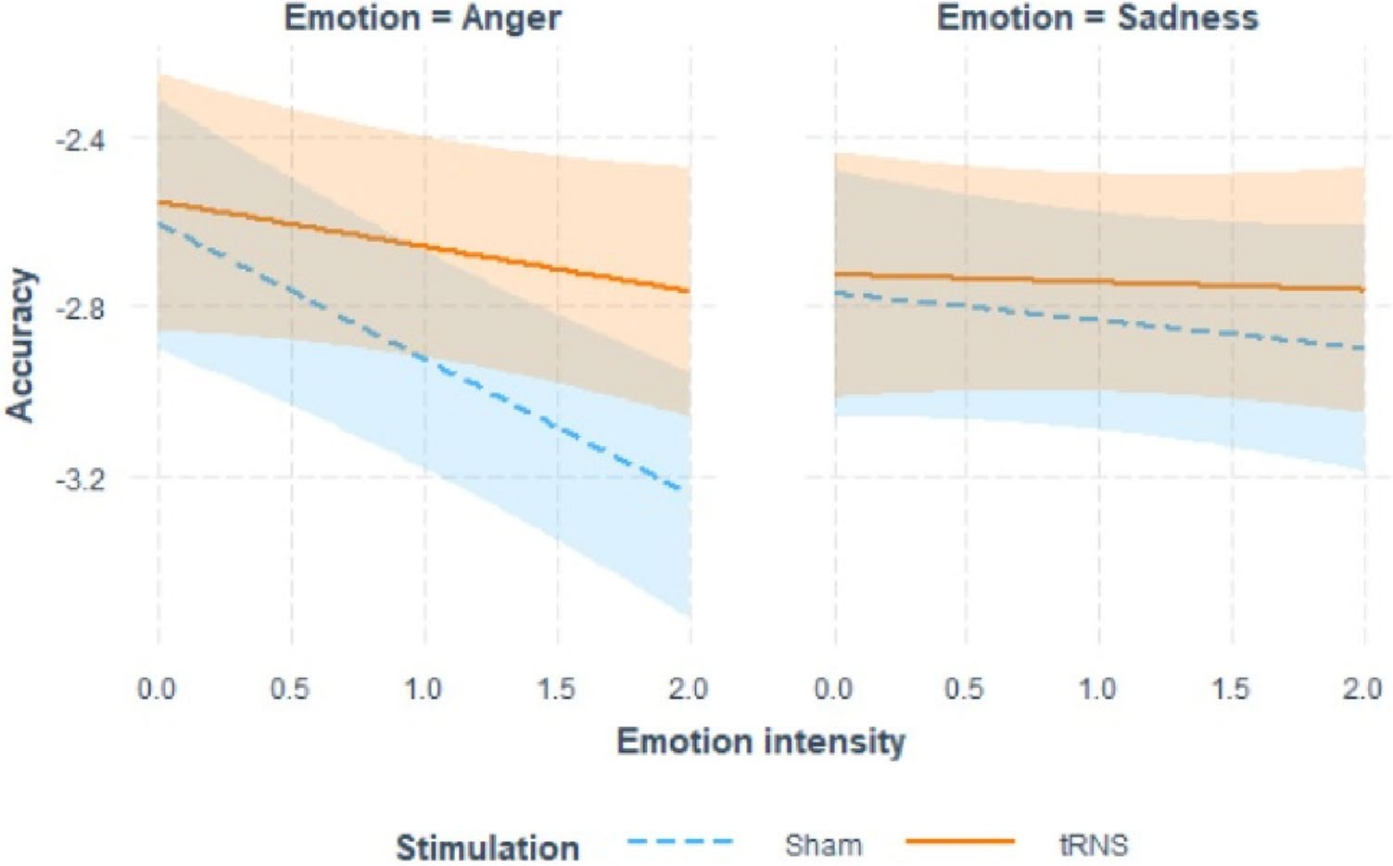
Interaction plot of the effects of emotion, emotion intensity and stimulation on iRT in the emotion recognition task. The figure shows the conditional effect of emotion intensity on iRT for sham (blue dashed lines) and tRNS (orange continuous line) stimulations separately for angry (left panel) and sad (right panel) emotional facial expressions. Shaded error bars indicate confidence intervals

Concerning the LMM on iRT, visual inspection of the residuals showed that they were skewed. As suggested by Baayen and Milin (2010), trials with absolute standardized residuals higher than 2.5 SD were considered outliers and removed (1.7% of the trials). After outlier trials removal, the model was refitted achieving reasonable closeness to normality. LMM results are presented in Table 3. We found significant Emotion × Intensity and Intensity × Stimulation interactions. As shown in Fig. 3, iRT decreased with increased emotion intensity. This effect was more pronounced for angry facial expressions and was reduce in the tRNS condition.

**Table 3:** Summary output of the LMM on iRT in the emotion recognition task

Predictors	Estimates	CI	*p*
(Intercept)	−2.69	− 2.95 − −2.42	** < 0.001**
Emotion	0.08	−0.04 – −0.21	0.203
Emotion intensity	−0.19	−0.29 − −0.10	** < 0.001**
Stimulation	0.05	−0.12 – 0.21	0.571
Emotion × intensity	−0.13	−0.22 − −0.03	**0.010**
Emotion × stimulation	0.00	−0.16 – −0.17	0.965
Intensity × stimulation	0.13	0.01 – 0.25	**0.041**
Emotion × intensity × stimulation	0.08	−0.04 – 0.21	0.191

Bold *p*-values indicate statistical significance at α = 0.05

Concerning the GLMM analysis on the neutral faces, we found a significant bias in categorizing them as “sad” (Odds Ratio = 0.12, CI 0.05–0.28, *p* < 0.001), which was not significantly modulated by stimulation (Odds Ratio = 1.22, CI  0.73–2.05, *p* = 0.455).

### Temporal task

Table 4 reports descriptive statistics for the temporal discrimination task. A preliminary *t* test was conducted on PSE for neutral images comparing sham and active stimulation; no main effect of stimulation was found at neutral stimuli (*t* (19) = 0.92, *p* = 0.371).

**Table 4:** Descriptive statistics of the temporal discrimination task

Stimulation	Emotion	Intensity	Interval duration						
			300	400	500	600	700	800	900
Sham	Neutral		0.03	0.14	0.28	0.55	0.76	0.82	0.89
			(0.06)	(0.12)	(0.16)	(0.24)	(0.17)	(0.18)	(0.13)
	Anger	Low	0.05	0.11	0.31	0.62	0.74	0.84	0.91
			(0.09)	(0.12)	(0.12)	(0.14)	(0.15)	(0.15)	(0.12)
		Medium	0.06	0.11	0.27	0.58	0.76	0.87	0.91
			(0.12)	(0.14)	(0.15)	(0.21)	(0.19)	(0.17)	(0.12)
		High	0.06	0.11	0.28	0.55	0.72	0.84	0.93
			(0.09)	(0.10)	(0.17)	(0.21)	(0.18)	(0.16)	(0.07)
	Sadness	Low	0.03	0.12	0.26	0.49	0.76	0.87	0.89
			(0.07)	(0.13)	(0.13)	(0.21)	(0.20)	(0.18)	(0.14)
		Medium	0.05	0.07	0.33	0.54	0.76	0.85	0.89
			(0.09)	(0.09)	(0.19)	(0.24)	(0.19)	(0.14)	(0.11)
		High	0.05	0.10	0.31	0.59	0.76	0.85	0.93
			(0.10)	(0.13)	(0.19)	(0.18)	(0.21)	(0.18)	(0.09)
tRNS	Neutral		0.04	0.08	0.31	0.52	0.70	0.86	0.87
			(0.06)	(0.07)	(0.18)	(0.21)	(0.22)	(0.16)	(0.13)
	Anger	Low	0.03	0.11	0.29	0.55	0.70	0.86	0.85
			(0.07)	(0.14)	(0.21)	(0.23)	(0.20)	(0.14)	(0.16)
		Medium	0.08	0.09	0.32	0.52	0.75	0.88	0.91
			(0.13)	(0.08)	(0.19)	(0.24)	(0.21)	(0.11)	(0.11)
		High	0.03	0.14	0.26	0.54	0.76	0.89	0.87
			(0.07)	(0.15)	(0.20)	(0.24)	(0.16)	(0.15)	(0.14)
	Sadness	Low	0.03	0.07	0.25	0.54	0.73	0.86	0.90
			(0.04)	(0.08)	(0.15)	(0.21)	(0.21)	(0.12)	(0.10)
		Medium	0.03	0.11	0.24	0.54	0.72	0.84	0.89
			(0.06)	(0.12)	(0.17)	(0.24)	(0.23)	(0.15)	(0.14)
		High	0.03	0.10	0.27	0.51	0.69	0.85	0.87
			(0.08)	(0.15)	(0.19)	(0.21)	(0.18)	(0.13)	(0.15)

Mean proportions of “long” responses are reported along with standard deviations.

**Fig. 4:**
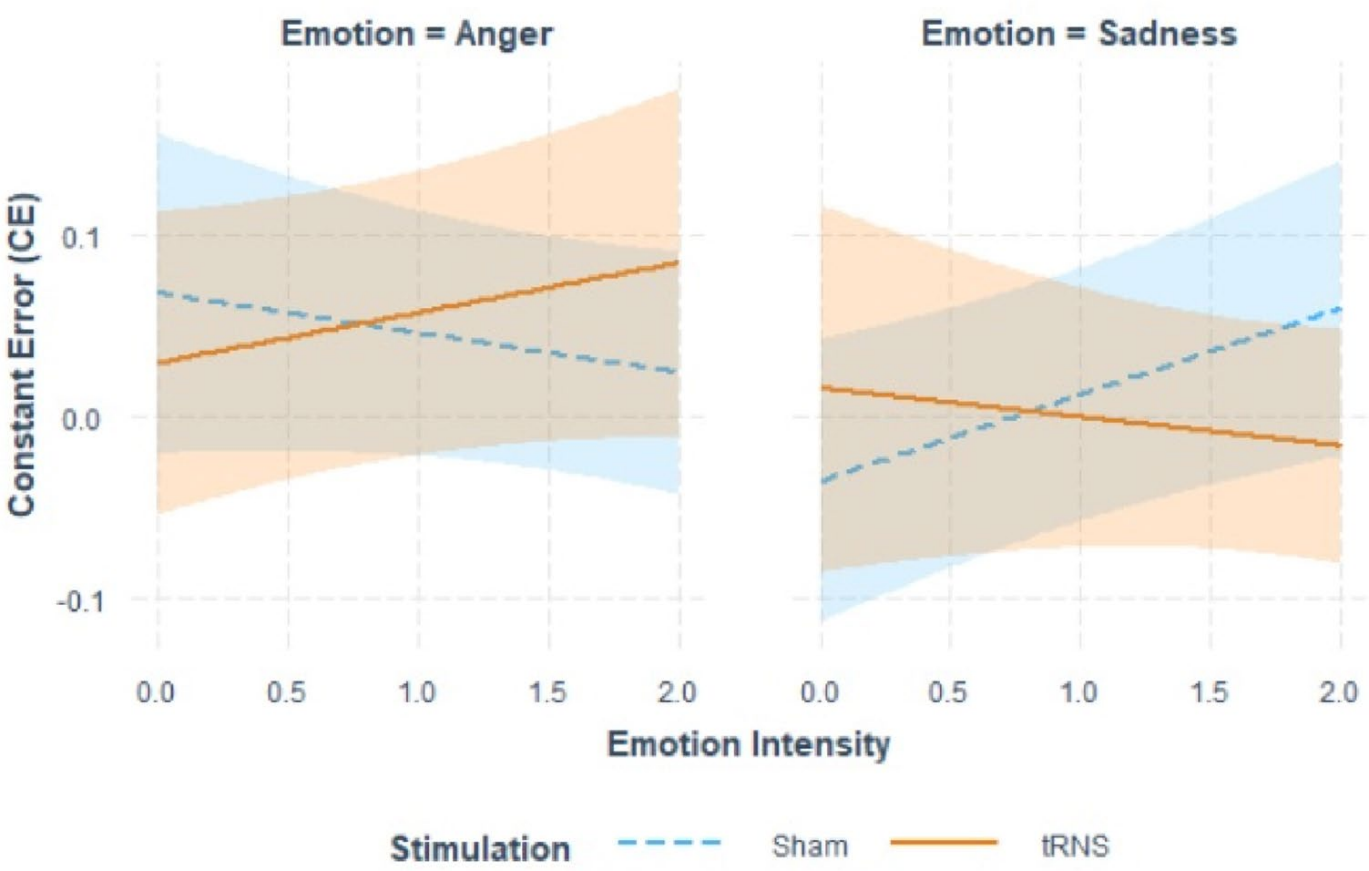
Interaction plot of the effects of emotion, emotion intensity and stimulation on Constant Errors (CE). The figure shows the conditional effect of emotion intensity on CE for sham (blue dashed lines) and tRNS (orange continuous line) stimulations separately for angry (left panel) and sad (right panel) emotional facial expressions. Shaded error bars indicate confidence intervals

The results of the LMM on CE are presented in Table 5. We found a significant Emotion × Intensity × Stimulation interaction. To further explore the three-way interaction, we conducted two additional LMM on CE separately for each of the two emotions. No significant effects were found for angry faces, while we found significant effects for Intensity (Estimate: 0.05, *p* = 0.018) and its interaction with Stimulation (Estimate: − 0.06, *p* = 0.049) for sad faces. As suggested in Fig. 4 (right panel) CE increased as a function of emotional intensity of sad faces, even if this effect was reduced in the tRNS condition. Concerning angry faces (Fig. 4, left panel), CE does not show any Intensity or Stimulation induced effects.

**Table 5:** Summary output of the LMM on constant error (CE)

Predictors	Estimates	CI	*p*
(Intercept)	0.02	−0.06 – 0.09	0.660
Emotion	0.05	0.01 – 0.09	**0.016**
Emotion intensity	0.01	−0.01 – 0.04	0.344
Stimulation	0.01	−0.11 – 0.12	0.916
Emotion × intensity	− 0.03	−0.06 − −0.01	**0.018**
Emotion × stimulation	− 0.04	−0.10 – 0.01	0.124
Intensity × stimulation	− 0.01	−0.05 – 0.04	0.758
Emotion × intensity × stimulation	0.06	0.02 – 0.09	**0.004**

Bold *p*-values indicate statistical significance at α = 0.05

### Sensation experienced questionnaire

No significant differences between tRNS and sham conditions were observed (Z = 0.26, *p* = 0.562; tRNS mean = 0.26, SD = 0.30; sham mean = 0.29, SD = 0.31).

## Discussion

We have all experienced how observing emotional stimuli can modify our relationship and experience of time. Here we investigated the effect of tRNS on emotion recognition and the subsequent effect on perceived duration of emotional face expressions. We expected temporal overestimation when participants were exposed to high-arousing emotional stimuli (faces expressing anger) compared to low-arousing stimuli (faces expressing sadness) and this overestimation being enhanced during active (random) stimulation.

Starting from the emotion recognition task, we observed that participants were more accurate in identifying the presented emotion as the intensity of that emotion increased. Beaupré and Hess (2005) and Dores and colleagues’ (2020) studies reported that sadness is usually better recognized than anger; in particular, Dores and colleagues (2020), using the same set of faces employed here, showed that sadness was 61.9% accurately identified compared with 55.9% for anger. When making the comparison, it should be considered that our task was a two-alternative forced choice, whereas theirs involved choosing between eight different types of emotions (anger, sadness, fear, disgust, surprise, happiness, contempt and neutral). Although the pattern of results described in Dores work (2020) seems consistent with what we observed here, we did not observe significant differences in recognizing the two emotions. Regarding the effect of stimulation on emotion recognition we targeted OFC because if its critical role in regulation of emotion (Dixon et al. 2017). The OFC is crucial for inferring the value of sensory objects based on contextual information including task structure (Jones et al. 2012; Stalnaker et al. 2014). The OFC is also interconnected with the amygdala and hypothalamus, and connections with the medial and lateral prefrontal cortex (Cavada et al. 2000; Petrides and Pandya 2007) may provide the OFC with information about social, task-related, and long-term goals. Thus, the OFC might act as a fulcrum in building a multidimensional representation of the current internal and external environment, to be exploited in emotional recognition and regulation as well as in goal directed behaviour. As a matter of fact, patients’ studies have shown that OFC damage is associated with an impaired ability to categorize or rate the intensity of facial expressions (Blair and Cipolotti 2000; Monte et al. 2013; Heberlein et al. 2008; Marinkovic et al. 2000). Typically, deficits reflect difficulty recognizing negative emotions (e.g., sadness, anger) as opposed to positive (e.g., happy) ones (Willis et al. 2014; see also Heberlein et al. 2008; Zald and Andreotti 2010, for reviews). In the present work, the analyses of RT and accuracy showed that emotion recognition improved with increasing in emotional intensity. Moreover, the RT analysis revealed an effect of OFC stimulation. Specifically, we observed faster RT as emotional intensity increased in the sham condition, while tRNS condition was associated with a less pronounced effect of emotional intensity. This result suggest that the stimulation of the OFC interfered with emotion recognition processes, and it is in line with previous studies indicating this area as part of the network involved in emotion recognition (Heberlein et al. 2008; Zald and Andreotti 2010).

Regarding the effect of emotion on timing, in line with previous studies (Droit-Volet et al. 2004; Gil and Droit-Volet 2011; Kliegl et al. 2015) we partially confirmed temporal overestimation when participants were exposed to high-arousing (angry faces) compared to low-arousing (sad faces) stimuli. According to the internal clock models (Gibbon et al. 1984; Treisman 1963; Zakay and Block 1995), this overestimation has been explained in terms of an increase in the arousal level induced by the perception of high-arousing stimuli, which in turns speeds up the internal clock mechanism underlying the representation of time. As suggested by Droit-Volet and Meck (2007) the temporal dilatation resulting from the speeding up of the internal clock might play as an adaptive mechanism allowing people to prepare more quickly in case of threatening events. However, in the present study, this overestimation was not always present, but modulated by stimulus intensity and by stimulation.

Starting from the effect of emotional intensity on temporal estimation, in the sham condition, participants tended to overestimate time when low-intensity angry faces were presented compared to low-intensity sad faces; the differences between sad and angry faces in the sham condition decreased with increasing intensity of the presented emotion. Concerning angry faces, this pattern of results seems to reflect a ceiling effect. The interpretations of this finding can be twofold. It might be that there was a limit to the extent to which emotional intensity could induce an increase in arousal and that this limit was already reached with low-intensity angry faces. Another potential explanation could be that there is a threshold to the degree at which arousal can prompt temporal overestimation. Subsequent investigations directly examining physiological responses linked to arousal could help to better explore these hypotheses. Concerning sad faces, temporal overestimation became evident with increasing in emotional intensity. The emotional intensity of face stimuli was taken as a proxy for arousal since previous evidence has shown a moderate to strong positive correlation between stimulus intensity and subjective arousal (Deckert et al. 2020). Therefore, findings for sad faces in the sham condition are in line with the hypothesis of temporal overestimation induced by arousing emotional stimuli. At the same time, the fact that at high intensity both emotions had the same effect on time is not in line with previous literature that indicated a clear overestimation of time when facial expressions of anger are used compared to the facial expression of sadness (Droit-Volet and Meck 2004; Droit-Volet et al. 2004, 2013; Fayolle and Droit-Volet 2014; Gil and Droit-Volet 2011). It should be noted that in absence of direct physiological measures it is difficult to speculate on this discrepancy. Moreover, Droit-Volet and Gil (2009) highlighted that there is great inter-individual variability in the sad face-related effect on time perception and, more recently, Colonnello and colleagues (2016) showed that the presentation of sadness was perceived as lasting shorter than that of other emotions (happiness, anger, disgust, or fear). Finally, other studies have shown that other aspects like attention or motivational direction influence the effects of sadness on time perception (Gable et al. 2016, 2022). The variety of observed effects of sadness on timing are puzzling and need further investigation.

When examining the effects of active stimulation of the OFC, differences between sad and angry faces also emerged. Specifically, in the case of sadness, the positive relationship between overestimation and emotional intensity was not evident under the tRNS condition. This aligns with what was observed in the emotion recognition task, suggesting that the stimulation likely interfered with OFC functions. This finding provides evidence for the potential role of the OFC in mediating the impact of emotionally arousing sad facial expressions on temporal estimation. However, this involvement is called into question when considering angry faces, as no significant effect of active stimulation was observed in this case. In the attempt to interpret this discrepancy, some speculations can be made. The stimulation of the OFC might have interfered with aspects specific to sadness, which influence time estimation, beyond just arousal. An example is motivation, which is known to be mediated by the OFC (Rolls 2023) and implicated in time perception (Gable et al. 2022). Sadness can be linked to both approach motivation (similar to anger) and withdrawal motivation. Research investigating the role of motivation in time perception has demonstrated that these types of motivation appear to have opposite effects on temporal estimation (Gable et al. 2022). In sum, although we found evidence supporting a distinct role of the OFC in mediating the effect of sad and angry facial expressions on time estimation, we are unable to draw definitive conclusions regarding the underlying mechanisms at this time.

In contrast with previous data (Mioni 2020; Mioni et al. 2018a, b), we did not observe temporal overestimation under tRNS compared to sham condition, but the overestimation was mediated by the emotional content of the stimuli presented. A direct comparison between the present findings and previous results is difficult because of the kind of considered stimuli (emotional stimuli here and neutral visual and auditory stimuli in Mioni et al. 2018a, b; Mioni 2020) and the targeted areas (OFC in the present study; parietal and frontal areas in Mioni et al. 2018a, b and primary auditory and visual cortices in Mioni 2020). It is important to note that here we targeted OFC, which is an area mainly involved in emotion recognition and emotional regulation (Bechara et al. 2000; Golkar et al. 2012; Willis et al. 2010) and temporal discounting (Sosa et al. 2021) rather than perceived duration (Mioni et al. 2020; Wiener et al. 2010).

We also acknowledge possible limitations of the present study. First, on the basis of previous literature, the emotional intensity of face stimuli was used as an indicator or proxy for arousal (Deckert et al. 2020). However, the fact that we observed a comparable effect of stimulus intensity between the two types of emotions in the discrimination task but not in the temporal task raises some questions along with a limit of the present study. It has been shown that there is no unique relationship between emotional discrimination and physiological responses (Folz et al. 2022), which differ depending on the emotional type. Since we do not have direct measures of physiological responses associated with arousal, our conclusions on the emotional distortion of time perception are limited. Another limitation is represented by the reduced number of trials per condition. Based on safety guidelines provided by our ethic committee we are encouraged to stimulate up to 20 min: we selected the number of trials to include in both tasks to fit the 20 min of stimulation, considering that the effect of stimulation is at maximum level under active stimulation, and it decreases after the active stimulation (Nitsche and Paulus 2000, 2001).

Moreover, we targeted the OFC because we were interested in modulating the effect of emotion recognition on subjective time perception. We opted for an excitatory protocol (tRNS) to enhance emotion recognition, although the results did not confirm our predictions. Future studies may consider stimulating other brain areas specifically involved in temporal processing (Wiener et al. 2010) or the right supplementary motor area (right SMA) and the junction of the right inferior frontal gyrus and anterior insula (IFG/AI) involved in both emotion recognition and time processing (Tipples et al. 2015).

In summary, our findings supported previous research indicating that emotionally arousing facial expressions result in time overestimation. However, it is noteworthy that the effects of emotional intensity and OFC stimulation differed for angry and sad facial expressions in terms of time perception but showed similar effects on emotion discrimination. This suggests the involvement of distinct mechanisms in the emotional distortion of time depending on the type of emotion. Further investigation is needed to explore this hypothesis in future studies.

## Data Availability

The data that support the findings of this study are available from the corresponding author upon request.
